# The big picture: exploring the metabolic cross-talk in cancer

**DOI:** 10.1242/dmm.036673

**Published:** 2018-08-24

**Authors:** Almut Schulze, Mariia Yuneva

**Affiliations:** 1 Department of Biochemistry and Molecular Biology, Theodor-Boveri-Institute, Biocenter, Am Hubland, 97074 Würzburg, Germany; 2 Comprehensive Cancer Center Mainfranken, Josef-Schneider-Str.6, 97080 Würzburg, Germany; 3 Oncogenes and Tumour Metabolism Laboratory, The Francis Crick Institute, 1 Midland Road, London NW1 1AT, UK

## Abstract

Metabolic reprogramming is now well established as one of the hallmarks of cancer. The renewed interest in this topic has spurred a remarkable advance in our understanding of the metabolic alterations in cancer cells and in the tumour microenvironment. Initially, this research focussed on identifying the metabolic processes that provided cancer cells with building blocks for growth or to prevent oxidative damage and death. In addition to providing detailed insight into the mechanisms by which oncogenic signalling pathways modulate metabolic processes, this research also revealed multiple nodes within the metabolic network that can be targeted for the selective elimination of cancer cells. However, recent years have seen a paradigm shift in the field of cancer metabolism; while early studies focussed mainly on the metabolic processes within a cancer cell, recent approaches also consider the impact of metabolic cross-talk between different cell types within the tumour or study cancer within the organismal metabolic context. The Review articles presented in this themed Special Collection of Disease Models & Mechanisms aim to provide an overview of the recent advances in the field. The Collection also contains research articles that describe how metabolic inhibition can improve the efficacy of targeted therapy and introduce a new zebrafish model to study metabolic tumour-host interactions. We also present ‘A model for life’ interviews: a new interview with Karen Vousden and a previously published one with Lewis Cantley that provide insight into these two leaders' personal scientific journeys that resulted in seminal discoveries in the field of cancer metabolism. In this Editorial, we summarise some of the key insights obtained from studying cancer metabolism. We also describe some of the many exciting developments in the field and discuss its future challenges.

## Abstract (precis)

**Summary:** The guest editors of this Special Collection discuss recent advances in the field of cancer metabolism, as well as the challenges faced as this exciting research progresses.

## Introduction

Altered glucose metabolism in cancer cells was discovered almost 100 years ago, when Otto Warburg demonstrated that tumours, instead of fully oxidising glucose to CO_2_, switch to aerobic glycolysis and ferment glucose to lactate (Warburg, 1924). Research over the past decade has greatly enhanced our understanding of metabolic reprogramming in cancer. It is now clear that the signals derived by oncogenes or tumour suppressors intersect with the metabolic network on multiple levels to drive the production of macromolecules for cancer cell growth and proliferation (Deberardinis et al., 2008). Moreover, cancer cells modulate their metabolic activity to cope with the unfavourable environmental conditions encountered within a tumour, such as nutrient deprivation and hypoxia. Experimental strategies to study cancer metabolism and analytical methods to identify the activity of metabolic pathways are getting more refined and have already provided an unprecedented insight into the wiring of the metabolic network. Similarly, the plethora of genetic information across different tumour types has revealed that metabolic enzymes drive cell transformation and tumour development (Vander Heiden and DeBerardinis, 2017). With this knowledge, researchers have developed viable treatment options targeting these drivers, adding to the arsenal of targeted cancer therapies (Waitkus et al., 2018).

## Finding new therapeutic targets

Targeting metabolism to treat cancer is not a new idea. Some well-used chemotherapeutic drugs, such as methotrexate, interfere with nucleotide biosynthesis to induce DNA damage and cell death in rapidly proliferating cells. Similarly, drugs that induce DNA damage or enhance oxidative stress in cancer cells also interact with their metabolism. The initial wave of studies investigating metabolic reprogramming in cancer focussed mainly on the metabolic processes that feed into biomass production. Cancer cells depend on these processes to support rapid growth and proliferation and, consequently, interfering with the components of these pathways reduces the ability of cancer cells to synthesize nucleotides, proteins or lipids. A clear disadvantage of therapeutic strategies targeting biomass accumulation is that they are likely to also affect proliferating normal tissues, such as the skin or the intestinal epithelium. One possible difference between these proliferating normal cells and cancer cells that could open a therapeutic window is the fact that biosynthetic processes compete with anti-oxidant pathways for reducing cofactors. As a consequence, cancer cells frequently increase oxidative damage in response to perturbations of the metabolic network (Schulze and Harris, 2012).

While the metabolic requirements of cancer cell proliferation are reasonably well understood, the analysis of cancer cell metabolism is still yielding some surprises. Metabolic pathways beyond the core glucose and glutamine metabolisms are receiving increasing attention. For example, inhibition of the urea pathway by deleting argininosuccinate synthase maintains aspartate pools for pyrimidine synthesis in cancer cells (Rabinovich et al., 2015). More recently, it was also shown that the repression of arginase 2 expression in renal cancer increases ornithine levels to suppress polyamine synthesis while promoting the production of pyridoxalphosphate, an essential cofactor for many biosynthetic reactions (Ochocki et al., 2018). The Review article by Keshet and Erez in this Special Collection of Disease Models & Mechanisms picks up this theme and discusses the potential roles for arginine and nitric oxide (NO) synthesis in cancer (Keshet and Erez, 2018).

Drugs targeting metabolic activities may also hold additional promise by synergising with existing targeted therapies in eliminating cancer cells. One of the Research Articles in this Special Collection provides an example. Lin et al. demonstrate that inhibition of choline kinase, an enzyme involved in the synthesis of phospholipids, can synergise with mTOR inhibition in combination with gemcitabine, an established chemotherapeutic agent (Lin et al., 2018).

## Zooming out – cancer and immune metabolism in the tissue context

While researchers explore the metabolic networks within cancer cells in ever more detail, recent years have witnessed a shift from a cell-autonomous perspective to a more integrated approach on cancer metabolism. This new holistic view integrates the internal metabolism of cancer cells with that of other cell types within the tumour microenvironment (Pavlova and Thompson, 2016). Metabolic coupling between cancer and stromal cells can provide essential nutrients that support cancer cell growth and survival. One example for this is provided by a study that demonstrated that pancreatic stellate cells, which form the stromal compartment of pancreatic cancers, secrete large amounts of alanine to support the metabolic activity of cancer cells (Sousa et al., 2016). Similar examples of metabolic coupling have been found for lactate, which is secreted by cancer-associated fibroblasts and supports the metabolism of breast cancer cells (Whitaker-Menezes et al., 2011), and for fatty acids, which are released from adipocytes and used for energy generation in metastatic ovarian cancer cells (Nieman et al., 2011).

In parallel to the detailed exploration of the metabolic activity of cancer cells, the metabolism of different immune cell types has been studied in great detail. This not only provided insight into the different metabolic programmes that immune cells engage during expansion, activation and differentiation (Puleston et al., 2017), but also revealed that tumour cells can utilise metabolic strategies for immune evasion. One of these strategies is metabolic competition, by which cancer cells deplete essential nutrients, such as glucose, from the tumour microenvironment and thereby prevent the activation of cytotoxic T cells (Chang et al., 2015). As the amino acid serine is an essential metabolite for effector T-cell expansion, local depletion of serine by cancer cells is also likely to impair immune cell infiltration and activation (Ma et al., 2017). Similar studies have also identified arginine and cystine as essential nutrients for effector T cells (Geiger et al., 2016; Siska et al., 2016). Conversely, cancer cells can secrete excess metabolites to create a hostile metabolic environment for immune cells. Indeed, the finding that lactate blunts immune surveillance by T cells and natural killer cells might finally provide a mechanistic explanation for the presumably wasteful aerobic glycolysis of cancer cells (Brand et al., 2016).

The ‘At a glance’ review and accompanying poster by Singer et al. provide an overview of the different microenvironmental changes and mechanisms of immunometabolic dysregulation and their links to cancer cell metabolism (Singer et al., 2018). It also highlights the challenges that the research community need to overcome to develop strategies by which metabolic competition or inhibition can be targeted to improve the efficacy of immune checkpoint inhibitors. In addition, the Review article by Hobson-Guiterrez and Carmona-Fontaine focuses on the metabolic determinants of macrophage polarisation in the context of the metabolic tumour environment (Hobson-Gutierrez and Carmona-Fontaine, 2018).

## Modelling the influence of the metabolic environment

As cancer cell metabolism is intricately linked to the local availability of nutrients and oxygen, targetable metabolic dependencies can only be identified under conditions that resemble the natural metabolic environment of cancer cells. Here, the field has seen a number of noteworthy developments that could revolutionise the way we culture cancer cells. Several recent studies have investigated the metabolic composition of human and mouse blood to determine the exact concentrations of different nutrients. This resulted in the development of adapted culture media that resemble physiological nutrient concentrations rather than being optimised for rapid cell growth at minimal cost. Metabolic dependencies identified *in vitro* using adapted media have a higher chance to be recapitulated in animal models (Tardito et al., 2015). Moreover, previously unrecognised differences in blood metabolite concentrations between mice and men have now been revealed, and could explain the differential efficacies of commonly used cytotoxic drugs. In this case, the high levels of uric acid found in human blood was shown to block the activity of uridine monophosphate synthase, which converts fluorouracil into fluorouridine triphosphate, the active compound that is incorporated into RNA instead of uracil to cause cell death (Cantor et al., 2017). Further refinement of media formulation and the introduction of three-dimensional tissue culture systems, such as organoids or spheroid cultures, and tumour-on-a-chip approaches (Sleeboom et al., 2018) will most likely reveal additional insight and improve the predictive power of *in vitro* systems.

However, given the complex metabolic cross-talk within the tumour microenvironment discussed above, it is clear that experimental models to study cancer metabolism should also recapitulate heterotypic cellular interactions. Here, advances have been made to analyse metabolic fluxes in live tumours using stable-isotope labelling techniques. One of these studies revealed striking differences in the metabolic activity of *Kras*-driven lung cancer cells cultured *in vitro* or studied in their natural tumour environment *in vivo* (Davidson et al., 2016). Surprisingly, lung cancer cells cultured *in vitro* displayed metabolic features of aerobic glycolysis and glutamine-dependent anaplerosis, while tumours generated by genetic induction of *Kras* or by implantation of murine or human lung cancer cells showed higher levels of oxidative glucose metabolism and no entry of glutamine into the tricarboxylic acid (TCA) cycle (Davidson et al., 2016).

Preclinical models are essential to develop and test potential therapeutic vulnerabilities of cancer cells, but many therapeutic strategies developed in these models ultimately fail in clinical testing. As demonstrated by the unexpected influence of uric acid on fluorouracil activation discussed above, the metabolic characteristics of a human tumour may not be adequately represented in a model organism. Here, advances have been made that allow the determination of cancer metabolism in human patients. Stable-isotope-labelled metabolites can be injected as a bolus prior to surgery or infused during surgery, and the excised tumour material can then be analysed by nuclear magnetic resonance and/or mass spectrometry (Fan et al., 2009; Hensley et al., 2016); these findings can also be integrated with diagnostic imaging modalities, such as contrast-enhanced magnetic resonance imaging, to determine tissue perfusion. In their seminal study, Hensley at al. used this technique to monitor glucose metabolism in human lung tumours and revealed substantial metabolic heterogeneity between patients but also between different areas within the same tumour (Hensley et al., 2016). Surprisingly, this study also found that highly perfused tumour areas mainly use alternative fuel sources, whereas less perfused areas revert to glucose for energy generation and anabolic metabolism. Although the exact reason for this metabolic adaptation is not fully understood, this finding could substantially affect treatment strategies aimed at blocking specific metabolic processes or at targeting the tumour vasculature.

The Review article by Muir et al. in this Special Collection describes the multiple microenvironmental factors that can contribute to the metabolic phenotype of cancer cells and discusses different approaches to improve existing experimental model systems (Muir et al., 2018). Moreover, the research paper by Enya et al. describes a zebrafish tumour model in which the metabolic cross-talk between tumours and the host liver can be studied (Enya et al., 2018).

## Dissecting tumour metabolic heterogeneity – emerging technologies

Together with the development of new models that closely recapitulate the natural environment of a tumour, as discussed above, dissecting the complex interactions between different cell types and environmental factors within tumours requires the development of new analytical methods. As discussed in the Reviews by Hobson-Gutierrez and Carmona-Fontaine (2018) and Muir et al. (2018), the challenge is to assess the metabolic state of individual cells while preserving tissue architecture and their temporal and spatial distribution. Several emerging techniques are taking on this task. Matrix-assisted laser desorption ionisation (MALDI) and desorption electrospray ionisation (DESI) mass spectrometry allow the spatial identification of metabolites (Greer et al., 2011). DESI, for example, has been used to map metabolic heterogeneity of tumours (Inglese et al., 2017). Although these platforms have been moving towards a single-cell resolution (Dueñas et al., 2017), this is not commonly reached. State-of-the-art secondary-ion mass spectrometry techniques allow visualisation of metabolites with subcellular resolution (Passarelli et al., 2017). These metabolite imaging modalities can potentially be used to identify metal-labelled antibodies (Angelo et al., 2014), which would allow the precise co-alignment of metabolites with cell lineage markers and the expression of metabolic enzymes in specific cells. Furthermore, combining mass-spectrometry imaging platforms with stable-isotope tracing could identify the spatial distribution of not only the metabolite levels, but also of the activities of individual metabolic pathways.

## You are what you eat: the effect of diet on cancer cell metabolism

Another recent trend in cancer metabolism research is to consider the impact of dietary nutrients. A number of studies have investigated the effect of controlled diets on tumour growth. After the importance of serine and glycine for cancer cell survival had been demonstrated *in vitro*, work in mice demonstrated that switching to a diet deficient in serine and glycine reduces tumour growth. However, this effect was only seen in some of the tumour models studied. A notable exception were *Kras*-driven pancreatic cancers, which can survive serine and glycine starvation by upregulating *de novo* serine synthesis (Maddocks et al., 2017). This suggests that the impact of restricted diets is determined by the combination of genetic drivers of a given tumour. This raises the problem that patients would need to be stratified based on adequate biomarkers and that tumour heterogeneity could lead to rapid development of resistance. Future studies testing the selective removal of nutrients, such as other non-essential amino acids, in multiple cancer models are likely to reveal additional metabolic dependencies in cancer, and will help to determine the feasibility of diet-based therapeutic strategies.

Interestingly, dietary intervention can also modulate the response to established cancer therapies. One example for this concept was demonstrated by a study showing that provision of lipids from the surrounding tissue blocks the effect of anti-angiogenic therapy on the growth of colorectal cancer cells (Iwamoto et al., 2018). In this system, high-fat-diet-induced liver steatosis augmented the protective effect, suggesting that diet can modulate therapeutic efficacy. More recently, it was shown that histidine degradation drains the pool of tetrahydrofolate in cancer cells and synergises with methotrexate, a drug that inhibits folate synthesis (Kanarek et al., 2018). Interestingly, in this study, dietary supplementation of histidine conferred sensitivity to low doses of methotrexate *in vivo*, suggesting that drug resistance could be overcome by an easy-to-implement dietary supplement. Dietary intervention can also alter the impact of cancer therapeutics on organismal metabolism. This was recently demonstrated by the finding that a ketogenic diet, which is low in carbohydrates but rich in proteins and fat, greatly increases the anti-tumour effect of inhibitors targeting PI3-kinase alpha (PI3Kα). On a normal diet, treatment with these drugs induces insulin tolerance, which results in the additional release of insulin by pancreatic β-cells. Circulating insulin then binds to the insulin receptor on the surface of cancer cells and dampens the effect of PI3Kα inhibition. Placing tumour-bearing mice on a ketogenic diet prevented the induction of insulin secretion and enhanced the anti-tumour effect of the inhibitor (Hopkins et al., 2018). As ketogenic diets have been used as part of weight-loss strategies and for the treatment of epilepsy, it would be quite easy to include such regimens into the next generation of clinical trials for cancer.

## Metabolic drivers of metastasis formation and cancer cell plasticity

The concept that different metabolic environments require specific adaptation is also supported by the recent findings elucidating the metabolic requirements of metastasis. For example, breast cancer cells metastasising to the lung utilise the high levels of pyruvate available in this organ for pyruvate-dependent anaplerosis to fuel their TCA cycle (Christen et al., 2016). Similarly, asparagine availability also promotes the formation of lung metastasis in breast cancer by promoting epithelial-to-mesenchymal transition (Knott et al., 2018). Here, the availability of asparagine in the diet was shown to be crucial for the biological effect. Interestingly, silencing of asparaginase only blocked metastasis under normal dietary conditions. However, when additional asparagine was added to the diet, depletion of asparaginase had no effect. Similarly, another study found that exposure to the metabolic environment of the liver prompted colon cancer cells to upregulate aldolase B (ALDOB), which enhanced fructose metabolism and allowed metastatic outgrowth (Bu et al., 2018). Silencing of *ALDOB* or a low-fructose diet reduced metastasis growth and extended animal survival, further illustrating the importance of dietary nutrient provision.

In addition to pyruvate and various amino acids, the availability of fatty acids can also drive metastasis initiation. It was shown that slowly proliferating metastasis-initiating cancer cells derived from oral squamous cell carcinomas depend on the fatty acid transporter CD36 (Pascual et al., 2017). This study also demonstrated that dietary fatty acids, in particular palmitate, promote the metastatic potential of cancer cells, a finding that has potentially wider implications when considering the high palmitate content of western diets. Pascual et al. further discuss the contribution of cancer cell metabolism to metastasis formation in their Review article in this Special Collection (Pascual et al., 2018).

Beside metastasis formation, disease relapse and tumour recurrence after therapy are major determinants of cancer-associated deaths. While the exact nature of cancer stem cells (CSCs) is still somewhat debated (Burclaff and Mills, 2018), it is clear that tumours consist of heterogenic cell populations with different traits affecting their ability for tumour initiation and potential for plasticity. Several studies have investigated the metabolism of CSCs in different systems. These studies revealed that CSCs from pancreatic cancers depend on mitochondrial metabolism (Viale et al., 2014; Sancho et al., 2015). However, as discussed in the Review by Peixoto and Lima in this Special Collection, this metabolic trait of CSCs may not be universal and may depend on tumour type and tissue context (Peixoto and Lima, 2018).

## Non-canonical roles of metabolic enzymes

Another important development in cancer metabolism research is the increasing number of metabolic enzymes for which non-canonical functions have been discovered. This is particularly prevalent for glycolytic enzymes, many of which function as modulators of transcription or translation and can directly couple metabolic activity to gene expression (Snaebjornsson and Schulze, 2018). Moreover, metabolic enzymes can modulate cellular signalling processes through direct protein-protein interactions, such as the binding of the glycolytic enzyme phosphofructokinase 1 to the transcription factor TEAD, which leads to the activation of YAP/TAZ signalling (Enzo et al., 2015). Conversely, the gluconeogenic enzyme fructose bisphosphatase 1, which is frequently depleted in clear cell renal cancer, binds to and inhibits the hypoxia inducible factor 1 (Li et al., 2014). Some metabolic enzymes that function as metabolite kinases can also phosphorylate proteins. One example for this is the 6-phosphofructo-2-kinase/fructose-2,6-biphosphatase (PFKFB3), a bifunctional enzyme that generates the allosteric regulator fructose-2,6-biphosphate via its kinase domain to promote glycolytic flux. PFKFB3 can also phosphorylate p27 to promote cell cycle progression (Yalcin et al., 2014). Moreover, a related enzyme, PFKFB4, was recently shown to phosphorylate the nuclear receptor coactivator 3 (also known as SRC-3) and promote its interaction with the transcription factor ATF4. Together, these factors drive the expression of pentose phosphate pathway enzymes and enhance nucleotide biosynthesis in breast cancer cells (Dasgupta et al., 2018).

The sheer number of metabolic enzymes for which non-canonical functions have been identified, discussed in the Review by Huangyang and Simon in this issue, emphasises the tight connection between cellular metabolism and multiple regulatory processes in the cell (Huangyang and Simon, 2018). Through this cross-talk, metabolic reprogramming in cancer can also affect seemingly unrelated cellular functions that drive the cancer phenotype.

## Concluding remarks: can metabolism be targeted for cancer therapy?

Given the vast amount of information that has now been assembled in the field of cancer metabolism, we have to take stock and consider the actual progress in improving cancer therapy. So far, only a small number of compounds have actually made it into the clinic. Two of these target the activity of the ‘metabolic oncogenes’, isocitrate dehydrogenases 1 and 2, which are frequently mutated in acute myeloid leukaemia and secondary glioblastoma. Other compounds are still in clinical trials, with varying success. The challenges that researchers face during clinical development of compounds targeting metabolic enzymes are illustrated by glutaminase 1 (GLS1) inhibitors, the efficacy of which has been difficult to demonstrate. However, similar to the lessons learned from other targeted therapies, it is evident that these compounds need to be matched with the right patient. Here, a recent study found a high degree of co-dependence of cancer cell lines on GLS1 and γ-glutamylcysteine synthetase, an enzyme of the glutathione biosynthesis pathway. This study also shed some light on the biomarkers that can be used to identify patients who are most likely to benefit from GLS1 inhibitors (Daemen et al., 2018).

Although much progress has been made in unravelling the metabolic network in cancer cells, there is still much to be learned about how different genetic drivers determine metabolic dependencies in the environmental context of a growing tumour and how these dependencies can be translated into viable therapeutic strategies. Applying the ever-growing analytical toolbox that allows us to study metabolism with an unprecedented precision to advanced experimental models will allow us to probe ever deeper into the multi-faceted metabolic interactions in cancer.
